# Mortality trends and high-risk subgroups in hematologic malignancy-associated pulmonary embolism: a population-based analysis with complementary clinical data

**DOI:** 10.3389/fonc.2026.1989633

**Published:** 2026-10-28

**Authors:** Xi Quan, Ping Zhang, Nan Zhang, Jing Wu, Shifeng Lou, Zhiming Luo

**Affiliations:** 1 Department of Hematology, The Second Affiliated Hospital of Chongqing Medical University, Chongqing, Nan’an, China; 2 Department of Hematology-Oncology, Chongqing University Cancer Hospital, Hematologic Oncology Intelligent Diagnosis and Treatment Engineering Research Center of Chongqing Education Commission of China, Chongqing, China

**Keywords:** hematologic malignancies, mortality, older patients, pulmonary embolism, real-world study, risk stratification, supportive care

## Abstract

**Objective:**

Pulmonary embolism (PE) is an important supportive care concern in patients with hematologic malignancies, particularly older adults with coexisting thrombotic, bleeding, comorbidity, and treatment-related risks. This study first characterized temporal and demographic patterns in U.S. population-level mortality records involving hematologic malignancies and PE, followed by a complementary single-center real-world analysis of 30-day all-cause mortality and potentially vulnerable age- and sex-defined subgroups.

**Methods:**

Using CDC WONDER data, we analyzed U.S. mortality records from 1999 to 2023 and calculated age-adjusted mortality rates (AAMRs), annual percentage changes (APCs), and average annual percentage changes (AAPCs). We also analyzed a single-center retrospective cohort to describe 30-day all-cause mortality according to age and sex.

**Results:**

Population-level mortality from hematologic malignancies declined from 1999 to 2023 (AAPC, −1.75%; 95% CI, −1.81% to −1.69%), whereas mortality records involving hematologic malignancies and PE increased, with a faster rise after 2017 (APC, 3.02%; 95% CI, 0.51%–5.58%). Higher mortality rates were observed among males, individuals aged ≥75 years, non-Hispanic Black individuals, residents of the Western United States, and nonmetropolitan populations. In the complementary clinical cohort, 30-day mortality was 30.77% among patients aged ≥75 years and 14.63% among those aged <75 years (P = 0.223), and 16.95% among males and 16.67% among females (P = 1.000).

**Conclusion:**

Although overall mortality among patients with hematologic malignancies decreased over the past two decades, mortality involving hematologic malignancies and PE remained an important supportive care concern with persistent demographic and geographic disparities. Complementary real-world observations showed a numerically higher 30-day all-cause mortality in older patients, although the difference was not statistically significant. These findings support careful clinical assessment of potentially vulnerable patients and individualized supportive care that considers thrombotic risk, bleeding risk, comorbidity burden, and underlying malignancy characteristics.

## Introduction

Pulmonary embolism (PE) is the most severe clinical manifestation of venous thromboembolism (VTE) and can be life-threatening, requiring prompt recognition and treatment (1–3). Cancer is an established risk factor for VTE, and thrombotic events substantially contribute to morbidity, mortality, and healthcare burden among patients with malignancies (4). Although extensive studies have investigated cancer-associated thrombosis, most available evidence has focused on solid tumors, whereas the population-level mortality burden and clinical outcomes involving PE among patients with hematologic malignancies remain insufficiently characterized (5–8).

The management of PE in patients with hematologic malignancies is particularly challenging because these patients often experience competing thrombotic and bleeding risks. Hematologic malignancies and their treatments may promote thrombosis through malignant hypercoagulability, inflammatory activation, treatment-related endothelial injury, and other disease-specific mechanisms. Conversely, thrombocytopenia caused by bone marrow involvement, cytotoxic therapy, hematopoietic stem cell transplantation, or immune-mediated mechanisms may substantially complicate anticoagulation decisions. In addition, several anticancer therapies, including antiangiogenic agents, second- and third-generation BCR-ABL inhibitors, immune checkpoint inhibitors, and immunomodulatory agents, may further influence thrombotic risk (9–13).

These competing risks are particularly relevant in older patients. Aging is associated with increased VTE incidence, higher prevalence of comorbidities, increased anticoagulation-related bleeding risk, and poorer short-term outcomes after acute PE (14–17). Sex differences have also been reported in the epidemiology and outcomes of hematologic malignancies, suggesting that demographic characteristics may provide clinically relevant information for identifying vulnerable populations (18–20).

Advances in diagnosis and disease-specific treatment have improved survival among patients with leukemia, lymphoma, and multiple myeloma (21). Meanwhile, population aging has increased the overall burden of hematologic malignancies worldwide (22). As more patients survive longer with hematologic malignancies, supportive care complications such as PE have become increasingly important. However, comprehensive evidence regarding long-term mortality trends involving hematologic malignancies and PE at the population level, as well as short-term outcomes after PE diagnosis in real-world clinical settings, remains limited.

Therefore, we integrated a population-level mortality analysis using CDC WONDER data with a complementary single-center real-world cohort analysis. The CDC WONDER analysis was performed to characterize temporal trends and demographic disparities in mortality involving hematologic malignancies and PE among U.S. adults from 1999 to 2023. The real-world cohort was used to provide complementary clinical observations regarding 30-day all-cause mortality after PE diagnosis, with exploratory analyses focusing on age, sex. Together, these analyses aimed to characterize population-level mortality trends involving hematologic malignancies and PE and to identify potentially vulnerable clinical subgroups requiring further investigation.

## Materials and methods

### Population-level mortality analysis

Using publicly available CDC WONDER data, we examined deaths among U.S. adults aged ≥25 years from 1999 to 2023 (23). Diagnoses were classified according to the International Classification of Diseases, 10th Revision (ICD-10). For the analysis of mortality involving hematologic malignancies and pulmonary embolism (PE), hematologic malignancy was recorded as the underlying cause of death and PE (I26) as a contributing cause. This analysis represents population-level mortality involving hematologic malignancies and PE. Records in which PE was the underlying cause and hematologic malignancy was contributory were not included. For the analysis of overall hematologic malignancy mortality, hematologic malignancy was recorded as the underlying cause of death. Hematologic malignancies included lymphoma (C81–C85), immunoproliferative neoplasms (C88), multiple myeloma (C90), and leukemia (C91–C95).

Deaths were stratified by sex, age, race/ethnicity, U.S. Census region, and urbanization level. Age was categorized into 10-year groups from 25 to 84 years, with a separate group for individuals aged ≥85 years. Regions were classified as the Northeast, Midwest, South, and West. Urbanization level was classified according to the 2013 National Center for Health Statistics Urban–Rural Classification Scheme for Counties. The urban–rural analysis was restricted to the period from 1999 to 2020.

### Study design and clinical cohort

As a complementary clinical analysis, we retrospectively included patients with confirmed hematologic malignancies and PE who were admitted to the Department of Hematology at the Second Affiliated Hospital of Chongqing Medical University between 2017 and 2023. PE was diagnosed by CT pulmonary angiography, lung ventilation/perfusion scanning, echocardiography, or based on clinical assessment. Patients with concurrent other malignancies or congenital coagulation disorders were excluded.

We collected age, sex, and 30-day all-cause mortality. Thirty-day outcome status was unavailable for 10 patients; analyses were therefore based on available cases. Other malignancy-related, laboratory, comorbidity, and PE-severity variables were not consistently available and were therefore not included in the comparative analyses. Because anticoagulation data were incomplete, mortality was not compared by anticoagulation status. Given the relatively small size of the single-center cohort, stratified analyses focused on age and sex, with patients aged ≥75 years classified as the older age subgroup. Thirty-day all-cause mortality was compared between age and sex groups as an exploratory analysis.

These clinical analyses were considered descriptive and exploratory. Race/ethnicity, U.S. Census region, and urban–rural classification were used only in the population-level mortality analysis and were not included in the clinical cohort analysis because they were not directly comparable with those in the local single-center setting.

### Statistical analysis

For the CDC WONDER analysis, age-adjusted mortality rates (AAMRs) per 100,000 population were calculated using the 2000 U.S. standard population. Age-specific crude mortality rates were used in age-group analyses. Temporal trends were assessed using the National Cancer Institute Joinpoint Regression Program, version 5.1.0, allowing a maximum of four joinpoints over the study period. Monte Carlo permutation tests were used to estimate annual percentage change (APC), average annual percentage change (AAPC), and their 95% confidence intervals (CIs), with P ≤0.05 considered statistically significant.

For the clinical cohort, categorical variables were compared using the chi-square test or Fisher’s exact test, as appropriate. A P value <0.05 was considered statistically significant. Given the limited sample size and the descriptive nature of the clinical cohort, subgroup comparisons were interpreted as exploratory rather than as evidence of independent prognostic effects.

CDC WONDER data are anonymized and publicly available; therefore, this component of the analysis did not require institutional ethics review. The retrospective single-center cohort was conducted in accordance with the applicable institutional ethics requirements.

## Results

### Population-level mortality

From 1999 to 2023, 1,384,189 deaths among U.S. adults aged ≥25 years were recorded with a hematologic malignancy as the underlying cause of death (**Table 1**), including 13,379 that also listed PE as a contributing cause (Supplementary **Supplementary Table S1**). Overall mortality from hematologic malignancies declined during the study period, with an AAPC of −1.75% (95% CI, −1.81 to −1.69) (**Figures 1A, C**). In contrast, mortality involving both hematologic malignancies and PE increased in two phases: the APC was 0.75% (95% CI, 0.17 to 1.33) from 1999 to 2017 and 3.02% (95% CI, 0.51 to 5.58) from 2017 to 2023 (**Figures 1B, D**). Mortality patterns varied across states (**Figure 2**).

**Table 1:** Absolute number of deaths and age-adjusted mortality rate (AAMR) of patients with malignant hematological diseases from 1999 to 2023, and their changing trends.

Characteristic	Deaths			AAMR		
	1999	2023	Percent change (%)	1999 (95% CI)	2023 (95% CI)	AAPC (95% CI)
Total	54999	55858	1.56	31.05 (30.79 to 31.31)	20.21 (20.04 to 20.38)	-1.75 (-1.81 to -1.69)*
Female	26046	23661	-9.16	25.16 (24.85 to 25.47)	15.39 (15.19 to 15.59)	-2.00 (-2.07 to -1.92)*
Male	28953	32197	11.20	39.54 (39.07 to 40.00)	26.41 (26.12 to 26.71)	-1.66 (-1.78 to -1.55)*
Northeast	11494	9531	-17.08	31.04 (30.47 to 31.61)	18.68 (18.30 to 19.07)	-2.12 (-2.33 to -1.91)*
Midwest	13928	12740	-8.53	33.18 (32.63 to 33.73)	22.01 (21.62 to 22.40)	-1.70 (-1.77 to -1.63)*
South	18980	21656	14.10	30.34 (29.91 to 30.77)	20.64 (20.36 to 20.92)	-1.64 (-1.72 to -1.57)*
West	10597	11931	12.59	29.78 (29.21 to 30.35)	19.12 (18.78 to 19.47)	-1.74 (-1.83 to -1.66)*
Hispanic	2295	4759	107.36	22.89 (21.89 to 23.90)	16.29 (15.81 to 16.77)	-1.44 (-1.59 to -1.29)*
NH Black	4820	5636	16.93	30.95 (30.07 to 31.84)	20.62 (20.07 to 21.18)	-1.55 (-1.65 to -1.45)*
NH White	46795	43123	-7.85	31.83 (31.54 to 32.11)	21.25 (21.05 to 21.46)	-1.66 (-1.72 to -1.60)*
NH Other	979	2230	127.78	18.23 (17.02 to 19.44)	11.48 (11.00 to 11.96)	-1.72 (-2.07 to -1.38)*
Metropolitan	44524	46887(2020)^†^	5.31	30.89 (30.60 to 31.17)	21.01 (20.81 to 21.20)(2020)^†^	-1.86 (-2.05 to -1.67)*(2020)^†^
Nonmetropolitan	10475	10099(2020)^†^	-3.59	31.75 (31.15 to 32.36)	23.06 (22.60 to 23.52)(2020)^†^	-1.57 (-1.65 to -1.49)*(2020)^†^
25-34 years	959	541	-43.59	2.39 (2.24 to 2.54)	1.19 (1.09 to 1.29)	-2.73 (-3.60 to -1.86)*
35-44 years	1909	973	-49.03	4.24 (4.05 to 4.42)	2.19 (2.05 to 2.33)	-2.88 (-3.06 to -2.70)*
45-54 years	3941	2119	-46.23	10.77 (10.44 to 11.11)	5.23 (5.01 to 5.46)	-3.12 (-3.45 to -2.79)*
55-64 years	7542	6295	-16.53	31.72 (31.00 to 32.43)	15.04 (14.67 to 15.41)	-3.03 (-3.18 to -2.88)*
65-74 years	14315	14007	-2.15	77.72 (76.45 to 78.99)	40.38 (39.71 to 41.05)	-2.66 (-3.03 to -2.29)*
75-84 years	18064	19580	8.39	147.76 (145.61 to 149.92)	106.60 (105.10 to 108.09)	-1.34 (-1.49 to -1.19)*
85+ years	8269	12343	49.27	199.06 (194.77 to 203.35)	199.24 (195.73 to 202.76)	-0.17 (-0.30 to -0.05)*

^*^ indicates that p ≤ 0.05, which is statistically significant.

^†^ Urban-rural classification data were available only through 2020. Therefore, for the Metropol itan and Nonmet ropolitan categories, endpoint deaths, AAMR, percent change, and AA PC were calculated using data from 1999 to 2020.

**Figure 1:**
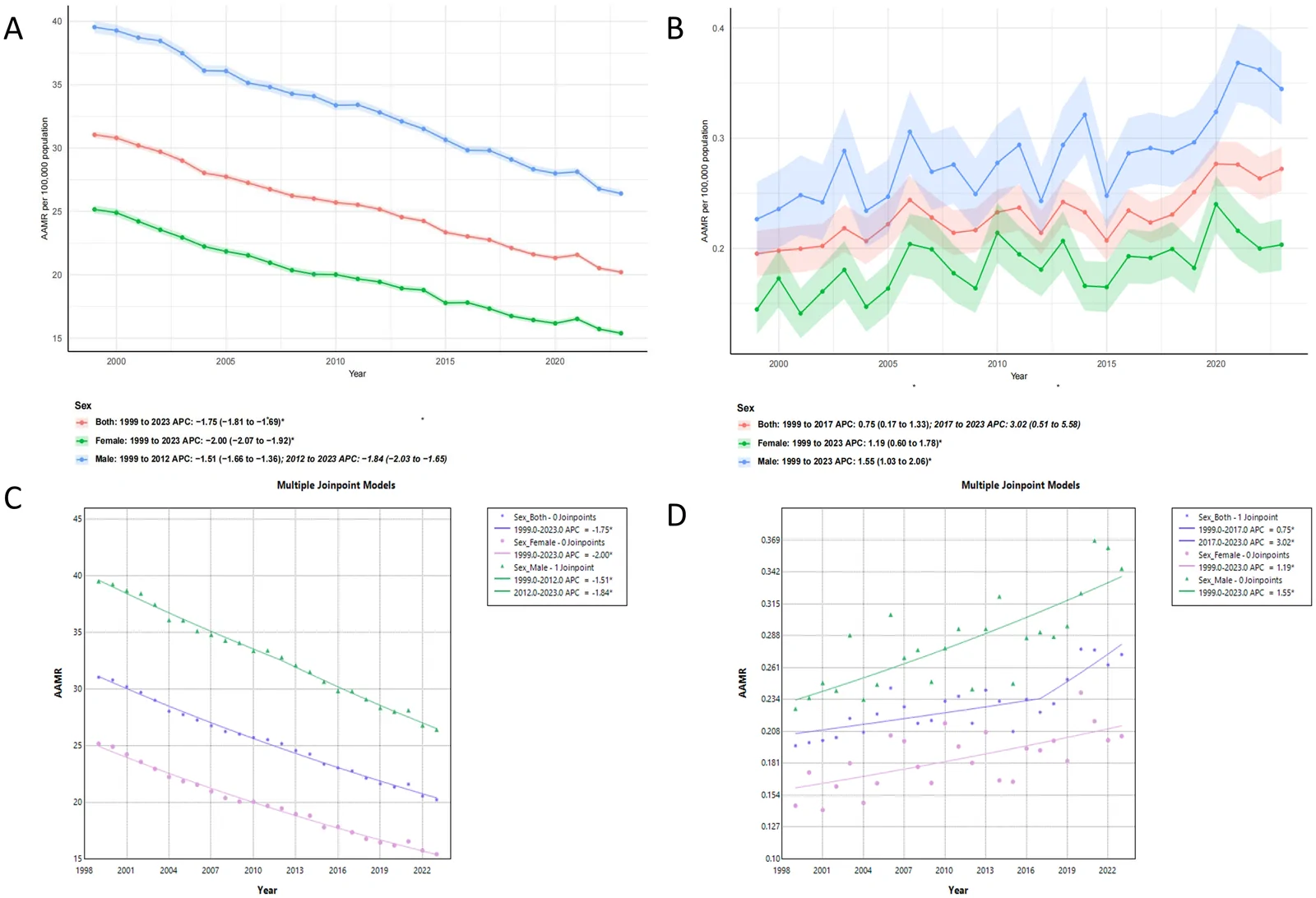
Age-adjusted mortality rates per 100,000 individuals for hematological malignancies [**(A, C)** joinpoint model] and hematological malignancies complicated by pulmonary embolism [**(B, D)** joinpoint model], stratified by sex, 1999-2023 (*indicates the APC is statistically significant; APC, annual percentage change).

**Figure 2:**
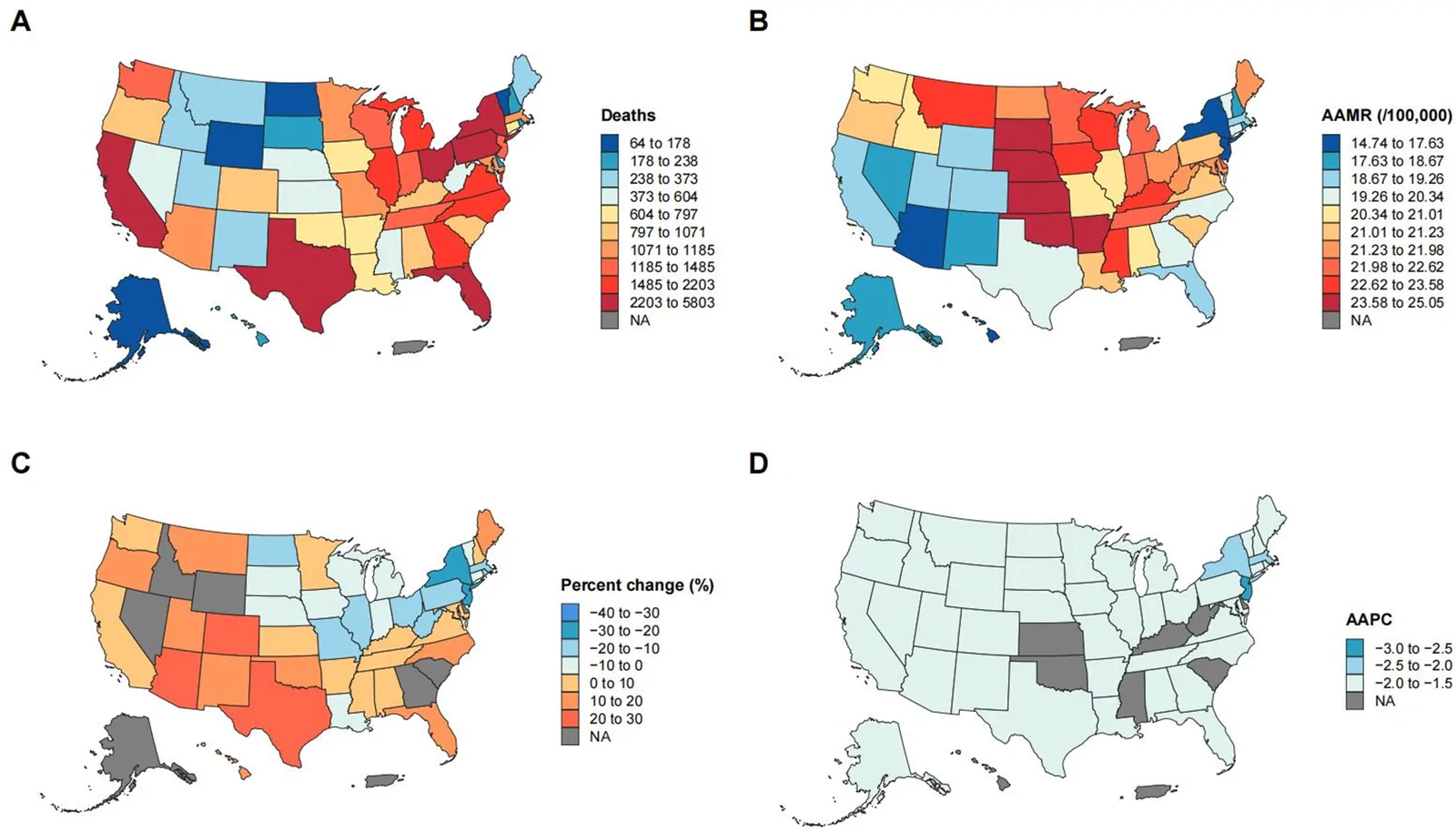
Absolute number of deaths **(A)**, age-adjusted mortality rates(AAMR) **(B)**, percent change **(C)**, average annual percentage change (AAPC) **(D)** of hematologic malignancy, stratified by state, from 1999 to 2023.

### Results by sex

Among records listing a hematologic malignancy as the underlying cause of death, 769,105 deaths occurred in males and 615,084 in females (**Supplementary Table S2**). In males, the AAMR declined from 39.54 (95% CI, 39.07 to 40.00) in 1999 to 26.41 (95% CI, 26.12 to 26.71) in 2023, with an AAPC of −1.66% (95% CI, −1.78 to −1.55). In females, the AAMR declined from 25.16 (95% CI, 24.85 to 25.47) to 15.39 (95% CI, 15.19 to 15.59), with an AAPC of −2.00% (95% CI, −2.07 to −1.92) (**Figures 1A, C**).

Among death records involving both hematologic malignancies and PE, 7,327 deaths occurred in males and 6,052 in females (**Supplementary Table S3**). The AAMR in males increased from 0.23 (95% CI, 0.19 to 0.26) in 1999 to 0.34 (95% CI, 0.31 to 0.38) in 2023. In females, it increased from 0.14 (95% CI, 0.12 to 0.17) to 0.20 (95% CI, 0.18 to 0.23). The overall APC was 1.55% (95% CI, 1.03 to 2.06) in males and 1.19% (95% CI, 0.60 to 1.78) in females (**Figures 1B, D**).

### Results by age

Age-specific crude mortality rates for hematologic malignancies generally declined, although the magnitude and statistical significance of the decline varied across age groups (**Figures 3A, C**). In contrast, PE-related mortality showed a clear age gradient (**Figures 3B, D**). No significant changes in trend were identified in any age group (zero joinpoints), suggesting a monotonic trend over time (**Figure 3D**).

**Figure 3:**
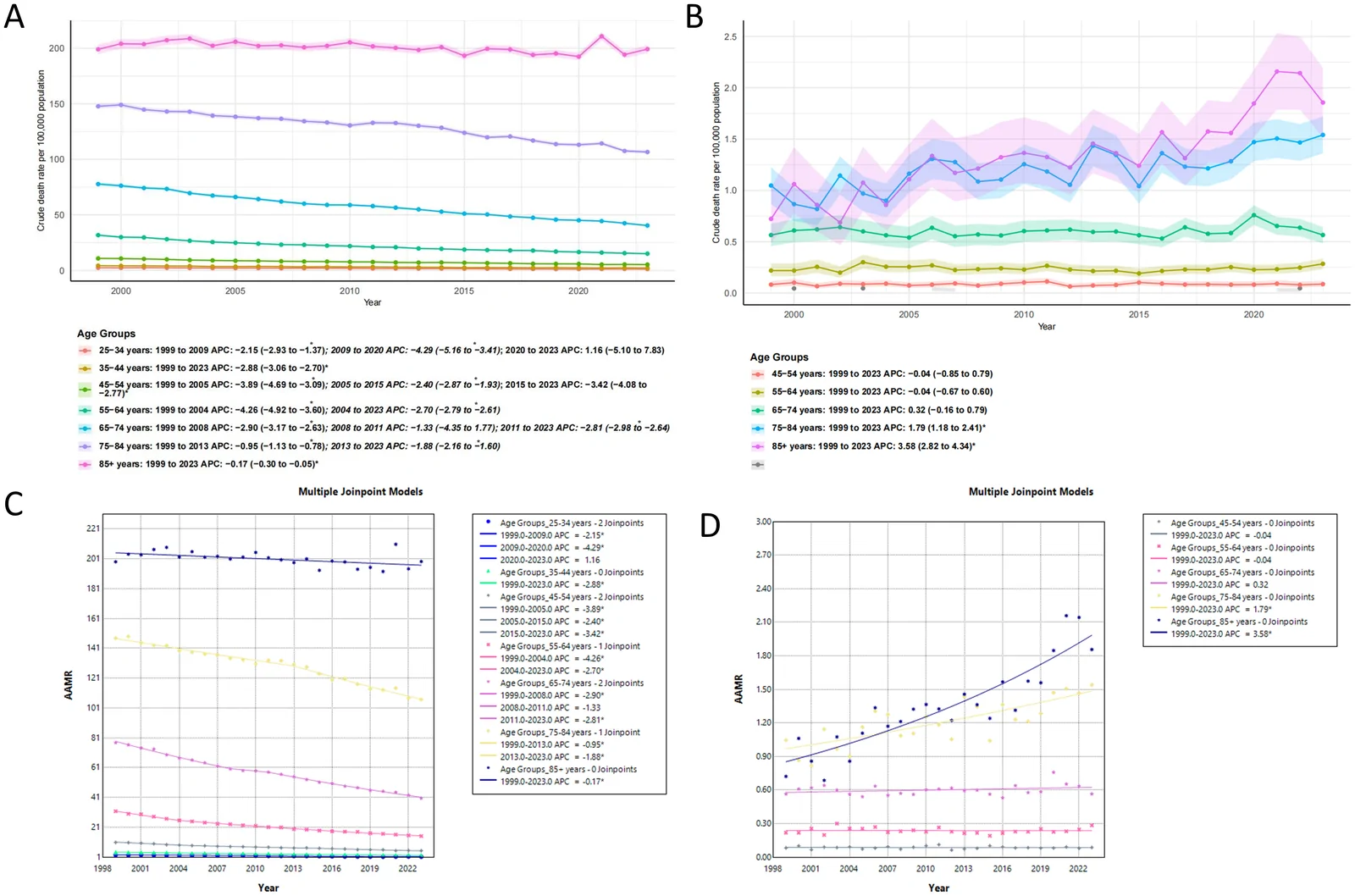
Crude mortality rate per 100,000 individuals for hematological malignancies [**(A, C)** joinpoint model] and hematological malignancies complicated by pulmonary embolism [**(B, D)** joinpoint model], stratified by age, 1999-2023 (*indicates the APC is statistically significant; APC, annual percentage change).

The APC was −0.04% (95% CI, −0.85 to 0.79) in those aged 45–54 years and 0.32% (95% CI, −0.16 to 0.79) in those aged 65–74 years; neither trend was statistically significant. Significant increases were observed in those aged 75–84 years (APC = 1.79%; 95% CI, 1.18 to 2.41) and ≥85 years (APC = 3.58%; 95% CI, 2.82 to 4.34).

### Results by region, urban–rural classification, and race/ethnicity

The overall AAMR for hematologic malignancies declined in all four U.S. Census regions (**Figures 4A, C**), whereas the PE-related AAMR increased in each region (**Figure 4B**). The APC for PE-related mortality was 1.06% (95% CI, 0.26 to 1.87) in the Northeast, 0.93% (95% CI, 0.14 to 1.74) in the Midwest, 0.84% (95% CI, 0.23 to 1.45) in the South, and 2.03% (95% CI, 1.44 to 2.62) in the West (**Figure 4D**).

**Figure 4:**
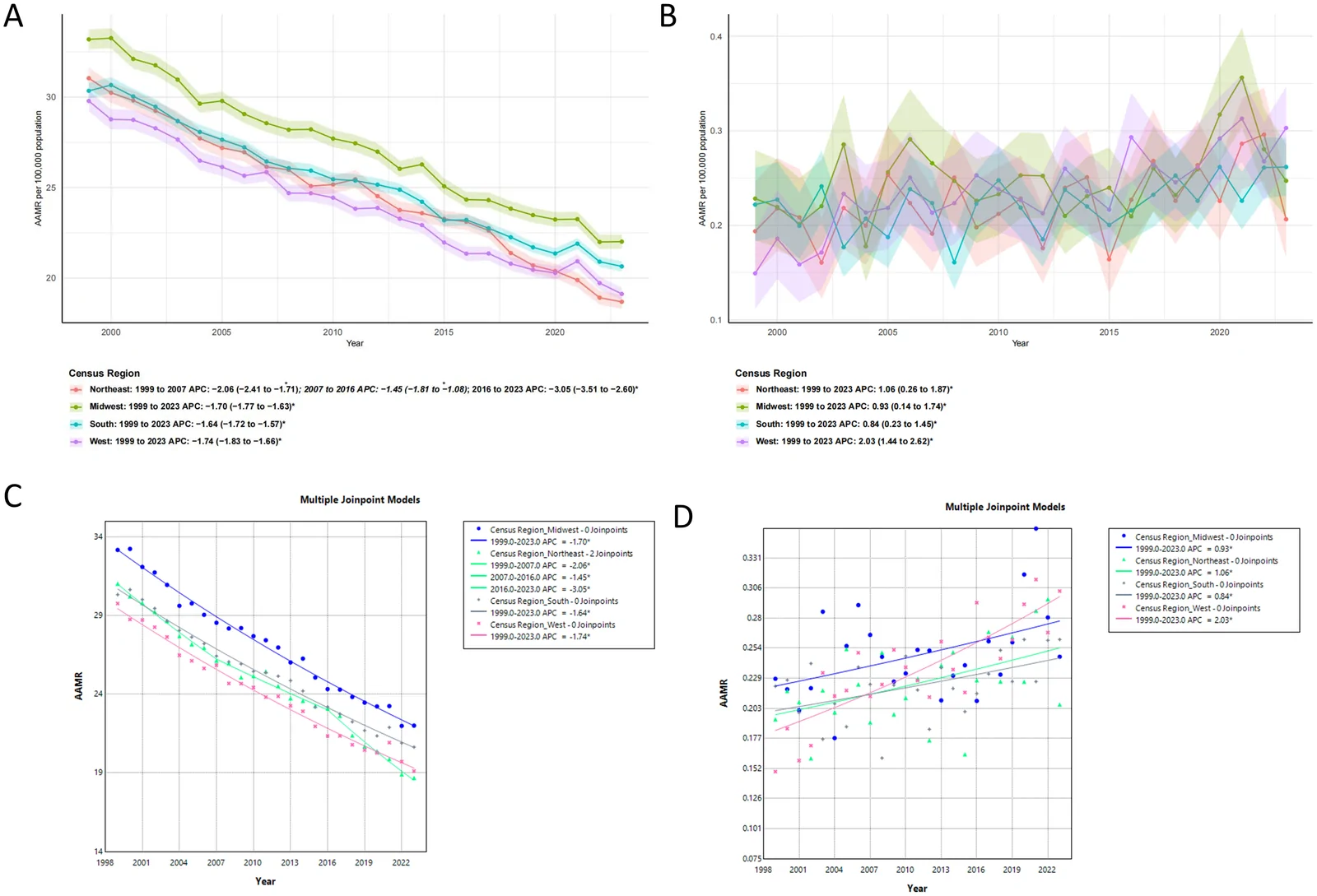
Age-adjusted mortality rates per 100,000 individuals for hematological malignancies [**(A, C)** joinpoint model] and hematological malignancies complicated by pulmonary embolism [**(B, D)** joinpoint model], stratified by region, 1999-2023 (*indicates the APC is statistically significant; APC, annual percentage change).

From 1999 to 2020, the overall AAMR for hematologic malignancies declined in both metropolitan and nonmetropolitan areas (**Figures 5A, C**), whereas the PE-related AAMR increased, with APCs of 0.89% (95% CI, 0.42 to 1.36) and 1.23% (95% CI, 0.24 to 2.22), respectively (**Figures 5B, D**).

**Figure 5:**
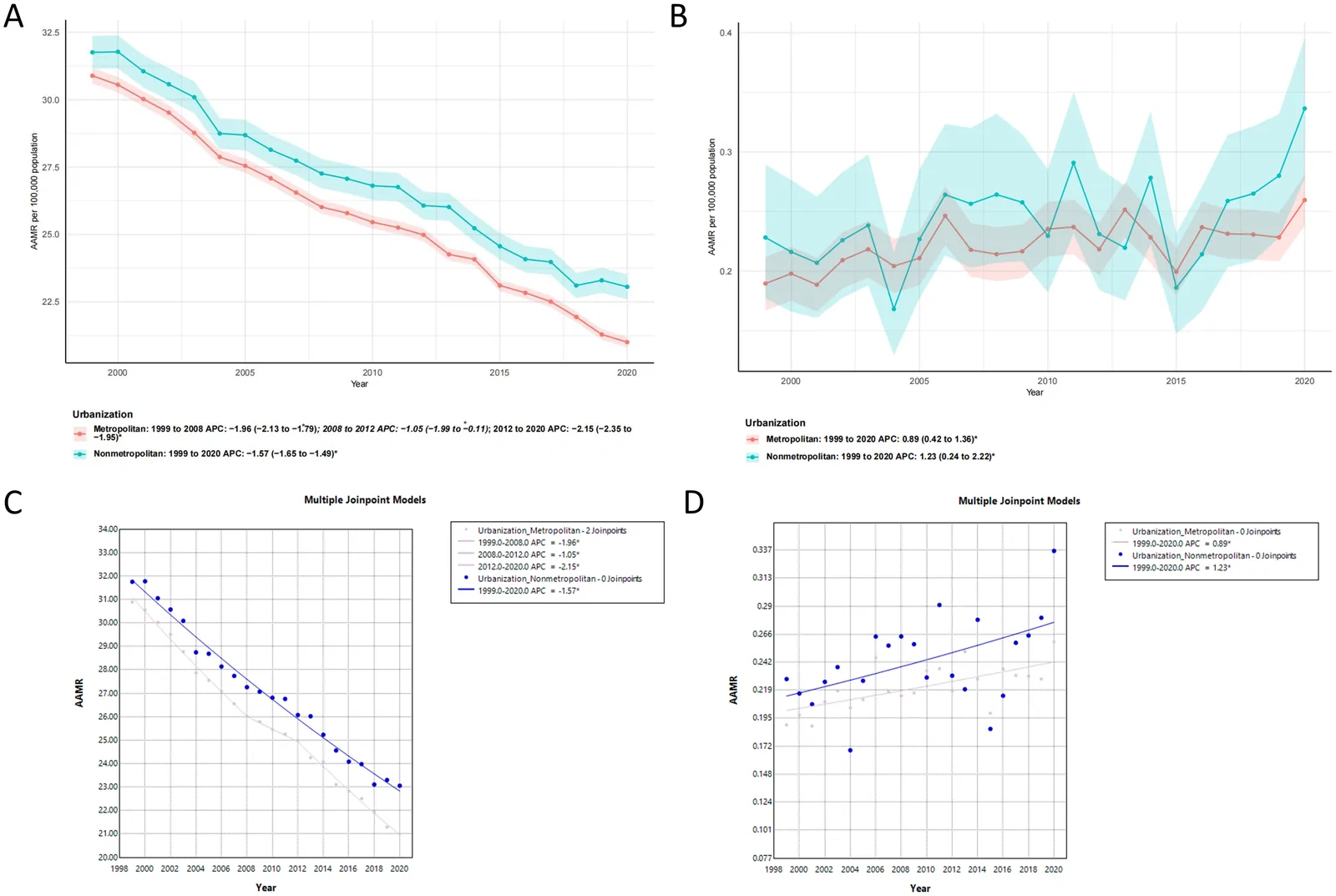
Age-adjusted mortality rates per 100,000 individuals for hematological malignancies [**(A, C)** joinpoint model] and hematological malignancies complicated by pulmonary embolism [**(B, D)** joinpoint model], stratified by urban vs. rural, 1999-2020 (*indicates the APC is statistically significant; APC, annual percentage change).

The overall AAMR for hematologic malignancies declined across racial/ethnic groups (**Figures 6A, C**). However, the PE-related AAMR increased in both non-Hispanic Black individuals (APC = 1.62%; 95% CI, 0.72 to 2.52) and non-Hispanic White individuals (APC = 1.59%; 95% CI, 1.12 to 2.07) (**Figures 6B, D**). Other racial/ethnic groups were excluded from the Joinpoint regression analysis because the available data were insufficient to estimate trends (**Figure 6D**).

**Figure 6:**
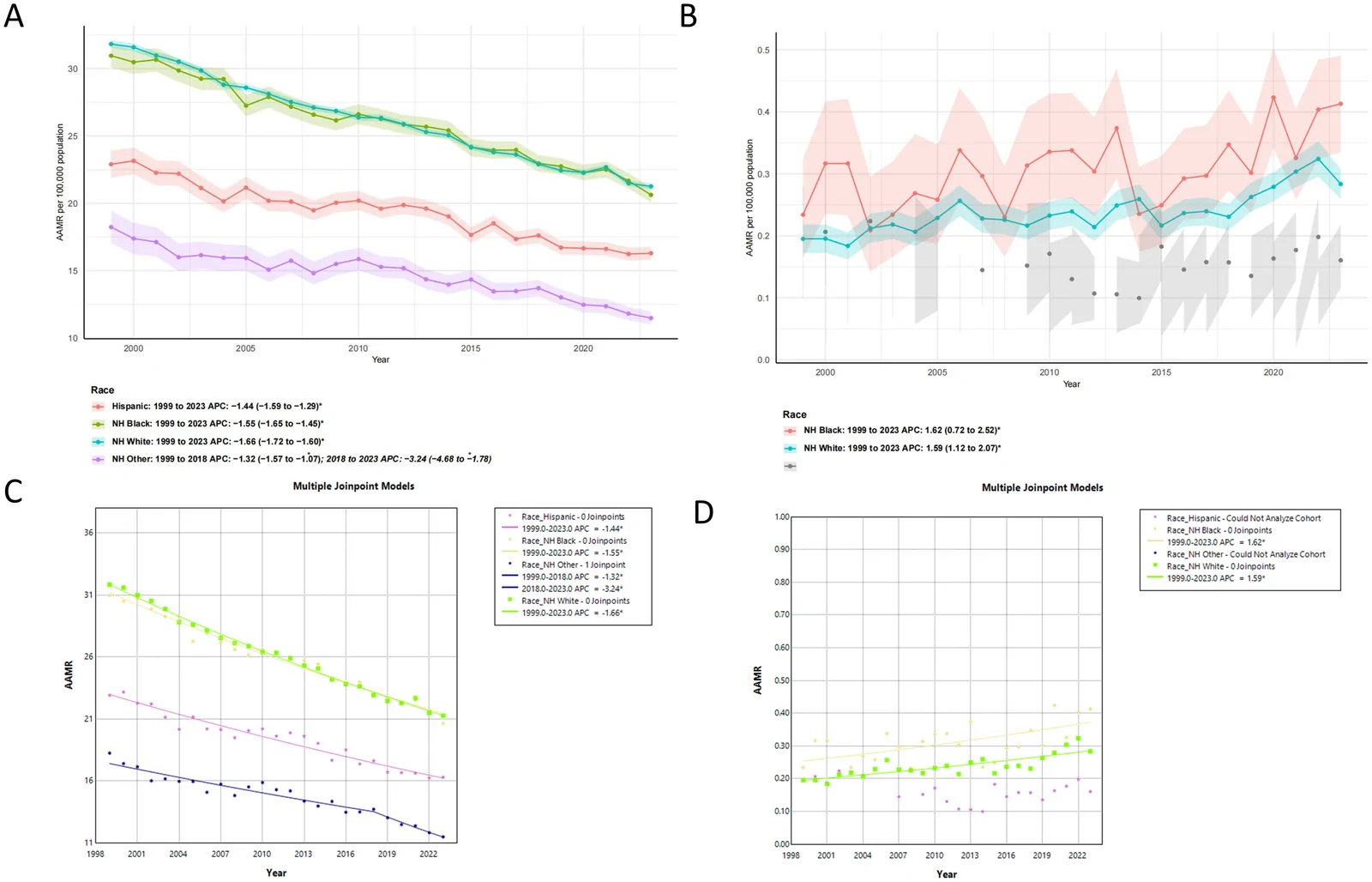
Age-adjusted mortality rates per 100,000 individuals for hematological malignancies [**(A, C)** joinpoint model] and hematological malignancies complicated by pulmonary embolism [**(B, D)** joinpoint model], stratified by race, 1999-2023 (*indicates the APC is statistically significant; APC, annual percentage change).

### Short-term mortality outcomes in the real-world cohort

We additionally assessed 30-day all-cause mortality in the single-center real-world cohort of patients with hematologic malignancies and PE. The original cohort included 105 patients. Because 30-day outcome status was unavailable for 10 patients, the mortality analyses were based on 95 evaluable patients, including 59 males and 36 females; 13 patients were aged ≥75 years and 82 were aged <75 years.

Among patients with available 30-day outcome data (n=95; 30-day survival status was unavailable for 10 patients), 30-day all-cause mortality was 30.77% (4/13; 95% CI, 9.09–61.43%) in patients aged ≥75 years and 14.63% (12/82; 95% CI, 7.80–24.17%) in those aged <75 years (**Figure 7A**). Although the difference was not statistically significant (P = 0.223), the numerically higher mortality in the older group suggests a possible signal of greater early clinical vulnerability that warrants further attention.

**Figure 7:**
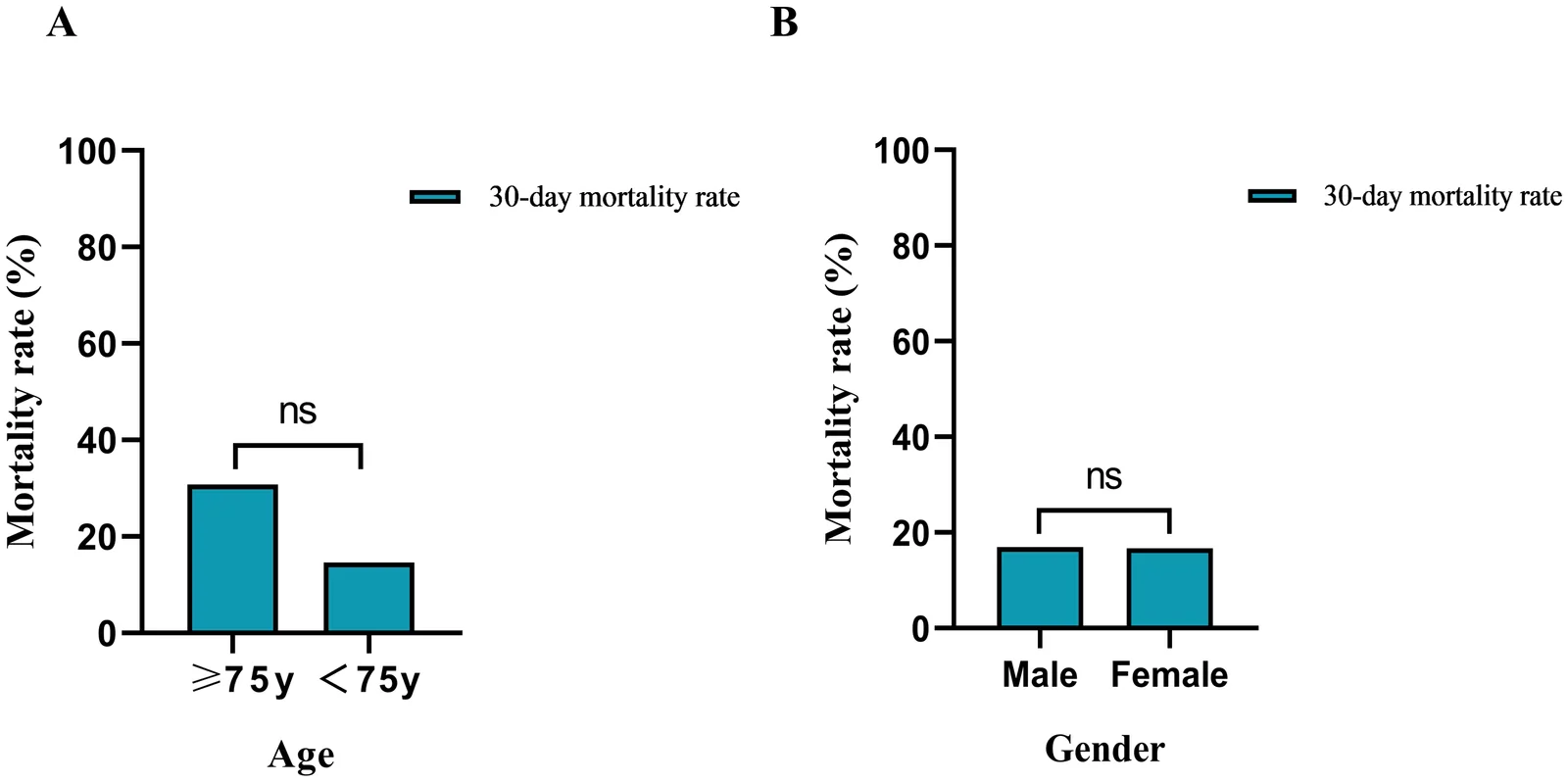
Thirty-day all-cause mortality by age and sex in the single-centercohort. **(A)** Mortality was 30.77% (95% CI, 9.09%-61.43%) in patients aged ≥75 years and 14.63% (95% CI, 7.80%-24.17%) in those aged <75 years (P=0.223). **(B)** Mortality was 16.95% (95% CI, 8.44%-28.97%) in males and 16.67% (95% CI, 6.37%-32.81%) in females (P=1.000). Between-group comparisons were performed using two-sided Fisher's exact tests. Confidence intervals were calculated using the Clopper-Pearson exact method.

Thirty-day all-cause mortality was 16.95% (10/59; 95% CI, 8.44–28.97%) in males and 16.67% (6/36; 95% CI, 6.37–32.81%) in females, with no significant difference between the groups (P = 1.000) (**Figure 7B**).

## Discussion

The population-level data showed that overall mortality from hematologic malignancies declined over the study period, whereas mortality involving both hematologic malignancies and pulmonary embolism (PE) increased, with a faster rise after 2017. Advances in diagnosis, treatment, and long-term follow-up may have contributed to the decline in overall mortality from hematologic malignancies (21, 22). In contrast, the increase in deaths involving PE may reflect a combination of the actual thrombotic burden, longer survival among patients with cancer, changes in treatment exposure, improved PE detection, and changes in death certificate coding. CDC WONDER cannot distinguish among these explanations. Nevertheless, the divergence between improving overall mortality from hematologic malignancies and increasing mortality involving both hematologic malignancies and PE indicates the continuing clinical importance of thrombotic complications.

The population-level analysis also demonstrated important age-related differences, with mortality involving hematologic malignancies and PE increasing more prominently in older age groups. Older age is directly relevant to PE management. VTE incidence increases markedly with age, while older patients face a higher risk of bleeding during anticoagulation and higher short-term mortality after PE (14–17). In patients with hematologic malignancies, these age-related vulnerabilities may coexist with thrombocytopenia, organ dysfunction, treatment toxicity, comorbidities, and reduced functional reserve. Consistent with this clinical vulnerability, the ≥75-year subgroup in our real-world cohort showed a numerically higher 30-day all-cause mortality, although the difference was not statistically significant.

Sex-related differences were also observed. Previous studies have reported sex differences in the disease burden or survival outcomes of hematologic malignancies (18–20). Public awareness of VTE remains limited (24), although the present study did not assess symptom recognition or delays in seeking care. The observed differences between males and females therefore warrant further investigation.

The mortality burden involving hematologic malignancies and PE was also higher in the western United States, nonmetropolitan areas, and non-Hispanic Black populations. Urban-rural mortality differences may be associated with healthcare access and socioeconomic conditions (25), and previous studies have also reported racial and ethnic differences in VTE incidence (26). However, CDC WONDER does not directly measure these mechanisms, and specific causal relationships therefore cannot be inferred.

The heterogeneous biological and treatment characteristics of hematologic malignancies further add to the complexity of thrombotic complications and supportive care. A comparative study of hospitalized patients suggested that PE presentation and outcomes may differ between patients with hematologic malignancies and those with solid tumors (27), while a population-based cohort study showed that VTE in patients with cancer was associated with poorer survival (28). Reductions in anticoagulant and fibrinolytic proteins associated with L-asparaginase may promote thrombosis (29), and lymphoma and acute leukemia also carry disease- and treatment-specific thrombotic risks (30–32). Cancer-associated coagulation is influenced by tissue factor and other procoagulant mechanisms, including those expressed by leukemic cells (33, 34). These differences suggest that a uniform approach may be inappropriate. Anticoagulation plans should account for the malignancy subtype, treatment phase, platelet count, PE severity, and individual bleeding risk.

In the complementary single-center real-world cohort, patients aged ≥75 years showed a numerically higher 30-day all-cause mortality than those aged <75 years, although the difference was not statistically significant. No meaningful difference was observed between males and females. Given the limited sample size and incomplete availability of several clinically relevant variables, the specific factors underlying these outcomes could not be determined. Nevertheless, the numerically higher mortality observed in older patients suggests a possible signal of greater clinical vulnerability that warrants further investigation.

Anticoagulation remains central to the management of PE, but treatment decisions in patients with hematologic malignancies must balance thrombotic and bleeding risks within the specific clinical context (2, 35, 36). This is particularly important because the presentation and outcomes of PE may differ between patients with hematologic malignancies and those with solid tumors (27), while VTE in patients with cancer is associated with poorer survival (28). Individualized management should therefore consider factors such as platelet count, bleeding risk, comorbidities, malignancy status, and PE severity.

Given the marked heterogeneity of hematologic malignancies, combining disease-specific molecular characteristics with readily identifiable clinical factors such as age and sex may help refine individualized risk assessment and prognostic stratification (37, 38). Studies in acute myeloid leukemia have also provided new insights into leukemia stem cell maintenance and treatment response (39, 40). As survival lengthens and treatment options expand, PE risk assessment and anticoagulation decisions also need to account for the underlying malignancy, treatment, and individual patient risk.

This study has several limitations. First, the clinical cohort was retrospective and derived from a single center, with a limited sample size and only 20 patients aged ≥75 years. Subgroup analyses were not adjusted using multivariable methods, and several clinically relevant variables were not uniformly available. The findings may therefore be subject to residual confounding by disease severity, contraindications to anticoagulation, treatment status, comorbidities, and PE severity. Several clinically relevant variables, including disease characteristics, bleeding risk, PE severity, and comorbidities, were not uniformly available, limiting cohort characterization and adjustment for potential confounding. Cause-specific mortality could not be consistently determined retrospectively; therefore, the observed 30-day all-cause mortality should not be interpreted as mortality directly attributable to PE. Second, CDC WONDER depends on the accuracy of death certificate coding and lacks individual-level information on malignancy subtype, platelet count, anticoagulation, cancer treatment, comorbidities, and PE severity. Finally, the U.S. population analysis and the Chinese single-center cohort differ in study setting and outcome definitions and therefore represent complementary components of the study.

Despite these limitations, this study links broader population-level mortality trends with clinically interpretable short-term outcome findings. Future multicenter studies with larger sample sizes and more complete clinical data are needed to further evaluate vulnerable subgroups and clarify the factors associated with short-term outcomes after PE in patients with hematologic malignancies.

## Conclusion

Despite the overall decline in mortality from hematologic malignancies, population-level mortality involving both hematologic malignancies and PE increased over time and showed persistent demographic and geographic disparities. In the complementary real-world cohort, patients aged ≥75 years showed a numerically higher 30-day all-cause mortality than younger patients, although the difference was not statistically significant, while no meaningful sex difference was observed. These findings underscore the continuing clinical importance of PE in patients with hematologic malignancies and support careful, individualized clinical assessment that considers thrombotic risk, bleeding risk, comorbidity burden, functional status, and underlying malignancy characteristics. Further multicenter studies with more complete clinical data are needed to clarify the factors associated with short-term outcomes and to inform more precise supportive care strategies.

## Data Availability

The datasets analyzed during the current study are available from the CDC WONDER database (https://wonder.cdc.gov/).
