# Supplementary material for: Mortality trends and high-risk subgroups in hematologic malignancy-associated pulmonary embolism: a population-based analysis with complementary clinical data
Source: Front Oncol. 2026 Oct 28;16:1989633. doi: 10.3389/fonc.2026.1989633 (PMC13751339; doi:10.3389/fonc.2026.1989633)
Supplement: Supplementary file 1 [file Table1.docx]

**Supplementary Material**

**Table S1. Absolute number of deaths and age-adjusted mortality rates (AAMR) of patients with hematological malignancies and pulmonary embolism from 1999 to 2023 and their changing trends. (* indicates that p ≤ 0.05, which is statistically significant)**

| Characteristic | Deaths | | |  | AAMR | | |
| --- | --- | --- | --- | --- | --- | --- | --- |
|  | 1999 | 2023 | Percent.change(%) |  | 1999 (95% CI) | 2023 (95% CI) | AAPC (95% CI) |
| Total | 366 | 766 | 109.29 |  | 0.20 (0.17 to 0.22) | 0.27 (0.25 to 0.29) | 1.31 (0.60 to 2.03)* |
| Sex |  |  |  |  |  |  |  |
| Female | 174 | 317 | 82.18 |  | 0.14 (0.12 to 0.17) | 0.20 (0.18 to 0.23) | 1.19 (0.60 to 1.78)* |
| Male | 192 | 449 | 133.85 |  | 0.23 (0.19 to 0.26) | 0.34 (0.31 to 0.38) | 1.55 (1.03 to 2.06)* |
| Census Region |  |  |  |  |  |  |  |
| Northeast | 72 | 105 | 45.83 |  | 0.19 (0.15 to 0.25) | 0.21 (0.17 to 0.25) | 1.06 (0.26 to 1.87)* |
| Midwest | 96 | 164 | 70.83 |  | 0.23 (0.18 to 0.28) | 0.25 (0.21 to 0.29) | 0.93 (0.14 to 1.74)* |
| South | 135 | 298 | 120.74 |  | 0.22 (0.18 to 0.26) | 0.26 (0.23 to 0.29) | 0.84 (0.23 to 1.45)* |
| West | 63 | 199 | 215.87 |  | 0.15 (0.11 to 0.20) | 0.30 (0.26 to 0.35) | 2.03 (1.44 to 2.62)* |
| Race |  |  |  |  |  |  |  |
| Hispanic | 19 | 48 | 152.63 |  | NA | 0.16 (0.12 to 0.22) | NA |
| NH Black | 40 | 117 | 192.50 |  | 0.23 (0.16 to 0.32) | 0.41 (0.34 to 0.49) | 1.62 (0.72 to 2.52)* |
| NH White | 299 | 580 | 93.98 |  | 0.20 (0.17 to 0.22) | 0.28 (0.26 to 0.31) | 1.59 (1.12 to 2.07)* |
| NH Other | NA | 19 | NA |  | NA | NA | NA |
| Urbanization |  |  |  |  |  |  |  |
| Metropolitan | 290 | 617(2020)† | 112.76 |  | 0.19 (0.17 to 0.21) | 0.26 (0.24 to 0.28)(2020)† | 0.89 (0.42 to 1.36)*(2020)† |
| Nonmetropolitan | 76 | 150(2020)† | 97.37 |  | 0.23 (0.18 to 0.29) | 0.34 (0.28 to 0.40)(2020)† | 1.23 (0.24 to 2.22)*(2020)† |
| Age Groups ^2^ |  |  |  |  |  |  |  |
| 25-34 years | NA | NA | NA |  | NA | NA | NA |
| 35-44 years | 14 | 10 | -28.57 |  | NA | NA | NA |
| 45-54 years | 30 | 35 | 16.67 |  | 0.08 (0.06 to 0.12) | 0.09 (0.06 to 0.12) | -0.04 (-0.85 to 0.79) |
| 55-64 years | 52 | 119 | 128.85 |  | 0.22 (0.16 to 0.29) | 0.28 (0.23 to 0.34) | -0.04 (-0.67 to 0.60) |
| 65-74 years | 104 | 196 | 88.46 |  | 0.56 (0.46 to 0.67) | 0.57 (0.49 to 0.64) | 0.32 (-0.16 to 0.79) |
| 75-84 years | 128 | 283 | 121.09 |  | 1.05 (0.87 to 1.23) | 1.54 (1.36 to 1.72) | 1.79 (1.18 to 2.41)* |
| 85+ years | 30 | 115 | 283.33 |  | 0.72 (0.49 to 1.03) | 1.86 (1.52 to 2.20) | 3.58 (2.82 to 4.34)* |

**† Urban–rural classification data were available only through 2020. Therefore, for the Metropolitan and Nonmetropolitan categories, endpoint deaths, AAMR, percent change, and AAPC were calculated using data from 1999 to 2020.**

**Supplemental Table S2.**Deaths by hematological malignancies and total deaths across the United States, stratified by gender 1999-2023.

| Year | Female Deaths | Female Population | Male Deaths | Male Population |
| --- | --- | --- | --- | --- |
| 1999 | 26046 | 94123092 | 28953 | 86285677 |
| 2000 | 26090 | 94864102 | 29057 | 87120538 |
| 2001 | 25724 | 95984408 | 29273 | 88320720 |
| 2002 | 25377 | 96927703 | 29550 | 89280325 |
| 2003 | 25101 | 97893297 | 29356 | 90197132 |
| 2004 | 24631 | 98921524 | 28792 | 91283860 |
| 2005 | 24550 | 100097839 | 29280 | 92453545 |
| 2006 | 24584 | 101328442 | 29269 | 93690917 |
| 2007 | 24305 | 102513830 | 29512 | 94889947 |
| 2008 | 24068 | 103688156 | 29789 | 96106934 |
| 2009 | 24058 | 104834186 | 30291 | 97272830 |
| 2010 | 24402 | 105717426 | 30161 | 98174557 |
| 2011 | 24445 | 107004480 | 31038 | 99588456 |
| 2012 | 24682 | 108088829 | 31359 | 100737208 |
| 2013 | 24481 | 109172782 | 31522 | 101912532 |
| 2014 | 24894 | 110581619 | 31720 | 103227661 |
| 2015 | 24029 | 111947362 | 31767 | 104606455 |
| 2016 | 24643 | 112990913 | 31637 | 105650504 |
| 2017 | 24473 | 114358026 | 32406 | 107089305 |
| 2018 | 24208 | 115265998 | 32538 | 108045192 |
| 2019 | 24226 | 116084432 | 32385 | 108896735 |
| 2020 | 24243 | 116909132 | 32743 | 109725881 |
| 2021 | 24096 | 116838617 | 32486 | 111399795 |
| 2022 | 24067 | 117317971 | 32024 | 112190628 |
| 2023 | 23661 | 118635845 | 32197 | 112893917 |
| Total | 615084 | - | 769105 | - |

**Supplemental Table S3.**Deaths by hematological malignancies complicated by pulmonary embolism and total deaths across the United States, stratified by gender 1999-2023.

| Year | Female Deaths | Female Population | Male Deaths | Male Population |
| --- | --- | --- | --- | --- |
| 1999 | 174 | 94123092 | 192 | 86285677 |
| 2000 | 186 | 94864102 | 191 | 87120538 |
| 2001 | 169 | 95984408 | 196 | 88320720 |
| 2002 | 193 | 96927703 | 203 | 89280325 |
| 2003 | 202 | 97893297 | 227 | 90197132 |
| 2004 | 177 | 98921524 | 215 | 91283860 |
| 2005 | 209 | 100097839 | 228 | 92453545 |
| 2006 | 231 | 101328442 | 270 | 93690917 |
| 2007 | 219 | 102513830 | 247 | 94889947 |
| 2008 | 201 | 103688156 | 255 | 96106934 |
| 2009 | 229 | 104834186 | 253 | 97272830 |
| 2010 | 251 | 105717426 | 266 | 98174557 |
| 2011 | 244 | 107004480 | 289 | 99588456 |
| 2012 | 240 | 108088829 | 253 | 100737208 |
| 2013 | 254 | 109172782 | 310 | 101912532 |
| 2014 | 233 | 110581619 | 331 | 103227661 |
| 2015 | 224 | 111947362 | 290 | 104606455 |
| 2016 | 263 | 112990913 | 330 | 105650504 |
| 2017 | 273 | 114358026 | 337 | 107089305 |
| 2018 | 283 | 115265998 | 331 | 108045192 |
| 2019 | 280 | 116084432 | 369 | 108896735 |
| 2020 | 373 | 116909132 | 394 | 109725881 |
| 2021 | 305 | 116838617 | 445 | 111399795 |
| 2022 | 322 | 117317971 | 456 | 112190628 |
| 2023 | 317 | 118635845 | 449 | 112893917 |
| Total | 6052 | - | 7327 | - |
